# Reducing waste in collection of quality-of-life data through better reporting: a case study

**DOI:** 10.1007/s11136-022-03079-1

**Published:** 2022-01-24

**Authors:** Victoria McCreanor, Elaine Lum, Nicholas Graves, Nan Luo, William Parsonage, Adrian Barnett

**Affiliations:** 1 AusHSI, Centre for Healthcare Transformation, Queensland University of Technology, Brisbane, Australia; 2 Jamieson Trauma Institute, Metro North Health, Herston, Australia; 3 Duke-NUS Medical School, Health Services & Systems Research, Singapore, Singapore; 4 Saw Swee Hock School of Public Health, National University of Singapore, Singapore, Singapore; 5 Cardiology, Royal Brisbane & Women’s Hospital, Herston, Australia

**Keywords:** Research waste, Health-related quality-of-life, Health utility, EQ-5D, Systematic reviews, Meta-analysis

## Abstract

**Purpose:**

This study describes the reporting of the preference-based health-related quality-of-life (HRQOL) instrument, the EQ-5D, and proposes strategies to improve reporting and reduce research waste. The EQ-5D is a validated instrument widely used for health economic evaluation and is useful for informing health policy.

**Methods:**

As part of a systematic review of papers reporting EQ-5D utility weights in patients with coronary artery disease, we noted the reasons data from some papers could not be reused in a meta-analysis, including whether health utility weights and sufficient statistical details were reported. Research waste was quantified using: (1) the percentage of papers and sample size excluded, and (2) researcher time and cost reviewing poorly reported papers.

**Results:**

Our search strategy found 5942 papers. At title and abstract screening 93% were excluded. Of the 379 full text papers screened, 130 papers reported using EQ-5D. Only 46% (60/130) of those studies reported utility weights and/or statistical properties enabling meta-analysis. Only 67% of included papers had reported EQ-5D in the title or abstract. A total sample size of 133,298 was excluded because of poor reporting. The cost of researcher time wasted estimated to be between $3816 and $13,279 for our review.

**Conclusions:**

Poor reporting of EQ-5D data creates research waste where potentially useful data are excluded from meta-analyses and economic evaluations. Poor reporting of HRQOL instruments also creates waste due to additional time spent reviewing papers for systematic reviews that are subsequently excluded.

**Recommendations:**

Studies using the EQ-5D should report utility weights with appropriate summary statistics to enable reuse in meta-analysis and more robust evidence for health policy. We recommend authors report the HRQOL instrument in the title or abstract in line with current reporting guidelines (CONSORT-PRO and SPIRIT-PRO Extensions) to make it easier for other researchers to find. Validated instruments should also be listed in the Medical Subject Headings (MeSH) to improve cataloguing and retrieval of previous research.

## Plain English summary

Being able to find and reuse published research is important for research progress, so time and money isn’t wasted repeating already completed experiments and so that new research can build on what is already known. Whilst researchers try to make their results available to others, sometimes the way scientific papers are written and catalogued makes it difficult to find and reuse previous research. This study highlights the difficulties researchers face when trying to find and reuse data from research on quality-of-life. We searched for research using the EQ-5D, a questionnaire about people’s health-related quality-of-life that is used in the evaluation of healthcare services. We wanted to use that previous research in an economic evaluation, rather than repeating a data collection. Because not all papers used the term EQ-5D, we searched for terms like “quality-of-life” to find relevant research. After reviewing thousands of papers, we found only about 2.2% [130/5942] had used EQ-5D, and less than half of those papers could be used for our research because of imperfect reporting. Our study shows that researchers could do better when reporting results of research using questionnaires like the EQ-5D. We suggest some simple ways to make it easier to find previous research including using the term “EQ-5D” in research summaries, and improving the way research papers are catalogued.

## Introduction

Chalmers and Glasziou estimated 85% of health and medical research is avoidably wasted with a large part due to biased or poorly reported results [1, 2]. This finding inspired the Lancet Series on increasing value and reducing waste in research, which made recommendations about how to improve reporting and reduce associated research waste [3–5].

Whilst the focus on reducing waste has tended to be on biomedical research and the field of psychology, the same concepts apply to other areas, including quality-of-life research. Patient-reported outcome measures (PROMs) including quality-of-life measures are not routinely collected in most healthcare settings or registries, and given the resources required to follow-up and collect data, it is unrealistic for many researchers to undertake bespoke collection of these data. This means that health services researchers often rely on secondary use of data collected during clinical trials or observational studies to inform economic evaluations [6]. Similarly, policy-makers may want to pool results from smaller studies, for a more comprehensive understanding of the quality-of-life of patients with a particular condition [7].

Cost-utility analysis, a type of cost-effectiveness analysis, uses health utility as the outcome of interest to assess the value of health services. Health utility is a measure of quality-of-life, between 0 (death) and 1 (full health), elicited using preference-based methods, the standard gamble or time trade-off, which require participants to make choices between different states of health. The most commonly used utility instrument is the EQ-5D, a questionnaire which asks participants to rate their health across 5 dimensions; mobility, self-care, usual activities, pain/discomfort and anxiety/depression. The combination of responses to the 5 questions is used to generate a health utility weight. Health economic modelling and evaluation often make use of previously published health outcomes data because they are not often able to be measured directly from the study population. Collating and synthesising these data generally requires a systematic, or reproducible, review with meta-analysis to ensure the best estimates of quality-of-life are used [8].

Systematic reviews rely on good reporting and cataloguing of studies by the original study teams for researchers to be able to find and reuse appropriate research. Quality-of-life and health utility measures are not often primary outcomes of trials and therefore may not be reported in abstracts of papers. This means it may not be evident from the title or abstract of a research paper that health utility was measured or what instruments were used. Whilst searches of clinical trial registries (e.g., clinicaltrials.gov) may help identify trials with quality-of-life measures listed as secondary outcomes, additional work is required to find the associated papers, if indeed all trial outcomes have been reported [9]. The CONSORT-PRO and SPIRIT-PRO Extensions to the reporting checklists for clinical trials aim to address this by encouraging researchers to report patient-reported outcomes consistently [10, 11]. An additional problem is that the phrase “quality-of-life” is generic, meaning it is sometimes used to describe improvements to health that were not measured using a validated instrument. For example, in the context of coronary artery disease, a reduction in chest pain symptoms is often described as an improvement to quality-of-life [12].

For researchers wanting to reuse health utility data, inadequate reporting of the instruments used makes the systematic review process more time-consuming because broad search criteria are needed to ensure potentially useful papers are captured, resulting in many papers not relevant to the meta-analysis topic also being captured and needing to be reviewed. Researcher time is thereby wasted, reviewing thousands of papers that end up being excluded from a review.

One way avoidable waste occurs is when potentially useful papers do not report health utility results in a format compatible with reuse in meta-analyses.

The aim for this paper is to examine two forms of avoidable research waste resulting from suboptimal reporting of quality-of-life data, relevant to systematic reviews and meta-analyses:

1. Research waste related to poor reporting of quality-of-life data, quantified as the proportion of papers reporting use of the instrument of interest, but not the statistical properties needed for reuse in meta-analysis, and,
2. Research waste in the form of researcher time, related to reviewing papers that do not fit the review criteria, retrieved due to the need to use broad search terms.

The context is a systematic review of papers reporting EQ-5D utility weights for people with coronary artery disease. We present recommendations for improving reporting, to reduce waste both in terms of researcher time to find and review studies, and to enable reuse of results for new research.

## Methods

The results in this paper relate to a systematic review of papers using EQ-5D to assess quality-of-life and health utility of patients with coronary artery disease. Briefly, we undertook systematic searches of literature databases, for papers published from January 2003 to March 2020, assessing the quality-of-life of patients with coronary artery disease, using the EQ-5D. The review protocol was published prior to data collection [13, 14]. The aim was to estimate the quality-of-life of patients with coronary artery disease at baseline and following different treatments including coronary artery bypass graft or percutaneous coronary intervention with or without stent, at 30 days, 6 months, 12–24 months and more than 24 months. To do this, we needed estimates of the EQ-5D utility weights, including mean and standard error, for the various patient groups and time points. Validated utility weights are generated using the participants’ responses across the five dimensions of the EQ-5D questionnaire. The EQ-5D visual analogue scale (VAS) has not been validated for this purpose, and therefore could not be used in our meta-analysis. The EQ-5D is available in two forms, EQ-5D-3L with three levels, or options, per question, and a five-level EQ-5D-5L form. For the purposes of this project, we included both the 3L and 5L versions.

We quantified two types of research waste associated with poor reporting of EQ-5D data collection and results: waste due to inability to reuse reported data in a meta-analysis, and waste of researcher time reviewing poorly reported papers.

## Researcher time and costs wasted reviewing poorly reported papers

We estimated the total time to review the title and abstract or full text of subsequently excluded papers using previous time estimates [15–17]. Because of the range of speeds of review reported by others, we created “fast” and “slow” reviewer scenarios, Table 1. The Cochrane Handbook for Systematic Reviews of Interventions suggests that title and abstract screening can be completed at a rate of 120 papers per hour [16]. Another study conducted a survey where the median hourly rate was 308 papers, with a maximum of 675 papers per hour [15]. We felt that the maximum reported rate of 675 papers per hour was unrealistic for most reviewers, so have used 308 for our fast scenario, Table 1.

**Table 1:** Papers screened per hour during review process

	Papers per hour	
	Slow	Fast
Retrieving full text documents*	15	30
Screen a title/abstract record	120	308
Screen the full text of a paper	12	24

*The “slow” rate was estimated based on Shemilt et al. [17]. We doubled that for the “fast” rate. Notably, the automated “find full text” function available in citation software (e.g., Endnote) has accelerated the time taken for retrieving full text papers. However, not all may be found this way perhaps due to limitations of the institution’s library subscriptions. Researchers may then use one or more manual methods to locate a full text copy of the paper—ResearchGate, Google Scholar, first author’s university repository, or requesting a copy from the authors of the paper. We propose these imperfect estimates for “slow” and “fast” taking into consideration the likely use of both the automated and manual methods for locating full text papers

We included the time wasted finding and cataloguing subsequently excluded full text documents for studies that could not be excluded at title and abstract screening. The rate at which full text documents were retrieved and screened for the “slow” scenario were taken from a cost-effectiveness study of systematic review methods [17]. We doubled those rates for the “fast” scenario. We calculated the time for two reviewers to screen each paper at each stage, as is standard practice for systematic reviews. We included the time for one reviewer to retrieve full text documents.

We calculated the costs of researcher time, using Queensland University of Technology salary scales for a mid-level professional staff (with a Bachelor degree and substantial research assistant experience) or an early to mid-career academic staff (with a PhD and some post-doctoral experience) in 2020 Australian dollars (Table 2) [18, 19].

**Table 2:** Hourly rates used to calculate wasted research costs

Salary scale	Hourly rate
Professional, research assistant (HEW 6.1)	$52.03*
Early to mid-career researcher (Level B.6)	$79.44**

*Casual rate

**Includes 30% on-costs, the costs associated with employing a staff member such as medical benefits and paid leave

## Research waste where results not able to be reused

At the full text screening stage, as per the 2009 and updated 2020 PRISMA statement and reporting guidelines [20], we noted the reason for excluding poorly reported papers. We estimated waste in terms of inability to reuse data as: the percentage of papers using EQ-5D but not reporting appropriate statistics to enable reuse of health utility data in a meta-analysis. Our meta-analysis required the mean EQ-5D health utility weight and standard error (SE) for each sample. Papers were excluded if they did not report those statistics, or other statistics enabling calculation of standard error; standard deviation (SD) and number of records. For example, papers which reported only median and interquartile range only were excluded. Papers reporting EQ-5D Visual Analogue Scale only or changes in health utility over time only were excluded. We calculated the percentage of research papers wasted using the number of studies not reporting reusable data and the total number of studies reporting use of EQ-5D.

We estimated the total sample size excluded due to poor reporting. We noted the sample sizes of all papers and estimated the amount of wasted data, using the total sample size excluded and the percentage excluded. Some studies measured EQ-5D at several times, so to avoid double counting we only included the study’s baseline sample. Where longitudinal data for the same cohort were reported over multiple papers, we only included the sample size at baseline from one paper, again to avoid double counting.

## Results

### Researcher time and costs wasted

**Fig. 1:**
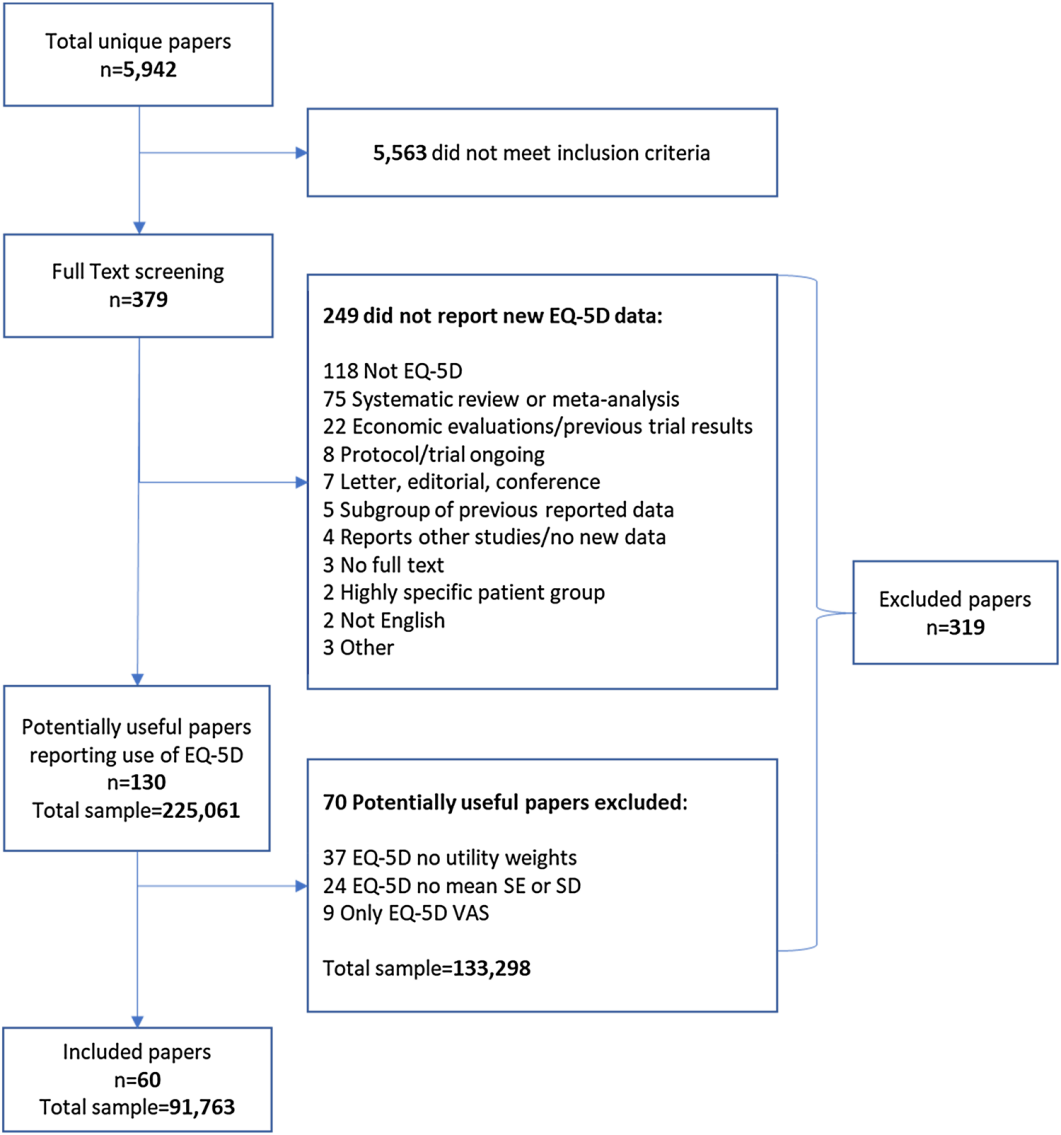
Flow chart of review process for inclusion in meta-analysis

A flow chart showing the review process is in Fig. 1. The number of papers reviewed and percentage of excluded papers at title and abstract, and full text screening stages are in Table 3.

**Table 3:** Overview of numbers of papers screened and excluded

	Total	Excluded (n)	Excluded (%)
Titles/Abstracts screened	5942	5563	94%
Papers for Full Text screening	379	319	84%

We excluded 94% of papers at title and abstract stage and 84% of full text papers. Overall, we found 60 papers could be used in our meta-analysis, just over 1% (60/5942) of the total papers reviewed. Of the 60 included papers, only 40 (67%) included the words EQ-5D or EuroQoL in the title and/or abstract, meaning the full text needed to be screened to identify which if any quality-of-life instrument was used.

Whilst title and abstract screening is relatively fast compared to full text screening, we estimated 36 to 93 h of researcher time was spent reviewing subsequently excluded papers, at a cost of between $1879 and $7366, Table 4. An additional 27 to 53 h were spent reviewing full text papers that were excluded, costing between $1379 and $4210, Table 4. We estimated that 88% to 89% of the total time spent screening abstracts, titles and full texts and searching for full text documents was spent on excluded papers, 73/83 h for the fast scenario and 167/187 h of the slow scenario, Table 4.

**Table 4:** Results of researcher time and costs wasted in fast and slow reviewer scenarios

	Fast			Slow		
	Time (hours)	Cost (low)	Cost (high)	Time (hours)	Cost (low)	Cost (high)
Total for all steps, all papers	83	$4308	$6578	187	$9754	$14,893
Excluded papers						
Searches for full text papers	11	$553	$845	21	$1107	$1689
Screening titles/abstracts*	36	$1879	$2870	93	$4824	$7366
Screening full text papers*	27	$1383	$2112	53	$2766	$4224
Total for excluded papers	73	$3816	$5826	167	$8697	$13,279

*Time calculations for screening are based on time for two reviewers

We screened the full text of 379 papers. The largest group of papers excluded at full text review were those that did not use an instrument to measure quality-of-life or used an instrument other than the EQ-5D, 37% (118/319) of excluded papers. If the instrument had been listed in the title or abstract, then this would have avoided 10 to 20 h of reviewer time ($512 to $1562).

## Research waste where results not able to be reused

We found a total of 130 papers reporting use of EQ-5D, but results reported in 70 (54%) were unable to be used in our meta-analysis, Table 5. If we recategorize the 9 papers that only used the EQ-5D Visual Analogue Scale (\* MERGEFORMAT Fig. 1) as not reporting EQ-5D, still only 50% (60/121) of papers were able to be reused. We found a total sample size across all studies reporting using EQ-5D of 225,061, 59% of which was excluded because we were unable to use the data reported in the paper (\* MERGEFORMAT Table 5).

**Table 5:** Research waste from papers reporting use of EQ-5D data

	Number of papers	Percent of total papers	Total sample size	Percent of total sample size
Total included	60	46%	91,763	41%
Total excluded due to poor reporting	70	54%	133,298	59%
Total reporting EQ-5D	130		225,061	

## Discussion

This case study of the process of systematically reviewing research highlights two ways in which research waste is generated due to poor reporting of methods and results. First, because the HRQOL instrument is often not reported in the title or abstract, systematic searches beyond the title and abstract are needed to retrieve potentially relevant papers, and their full texts need to be reviewed to correctly include or exclude them, creating waste in terms of researcher time. Secondly, waste is created when results are not reported in a way that enables reuse and/or meta-analysis.

Because of the first problem, poor reporting of HRQOL instruments, we spent many hours reviewing papers that were eventually excluded. Whilst we recognise that not all researchers reporting HRQOL measurement would anticipate reuse of their data in a meta-analysis, new trials should be set in the context of existing research [4] to avoid duplication of effort (another form of research waste), and therefore researchers should want their research to be easily discoverable and cited by others. Poor reporting also harms the original researchers when their results cannot be reused and the full impact of their work is not realised.

This first type of research waste could have been reduced and mostly avoided if other researchers had listed HRQOL instruments in their titles or abstracts, or if there were MeSH terms to capture them. Because one third (20/60) of included papers did not report the EQ-5D in the title or abstract, we needed to search for terms such as “quality-of-life”, meaning we captured many papers that used this term in a generic way but did not measure it. Additionally, whilst the EQ-5D measures preference-based health utility, that term is not often used outside of cost-utility analyses meaning potentially useful papers would be missed if we did not search for the term quality-of-life. Further, when it was clear from the abstract that HRQOL had been measured, for many papers, we needed to review the full text to know which instrument had been used. We reviewed the full text of 118 papers that used an instrument other than the EQ-5D, which we would not have had to review had the instrument been stated in the abstract. Whilst our research examined only use of the EQ-5D, we expect similar problems to arise when searching for research using other HRQOL instruments. The waste created here was researcher time spent reviewing papers that could have been more quickly included or excluded had reporting been better.

As a result of the broad search strategy needed to capture papers that did not report the HRQOL instrument in the title or abstract, we found that only 1% (60/5942) of papers we reviewed could be included in our meta-analysis and estimated the cost of researcher time wasted to be between $3816 and $13,279 for our review. Had we used a more limited search strategy, for example restricting the search only to papers which reported the EQ-5D in the title and/or abstract, we would have missed one third of the eventually included papers.

Whilst there will always be some time spent reviewing papers that are eventually excluded, excessive waste of researcher time can be avoided. Guidelines exist for reporting patient-reported outcome measures [10, 11], however it is clear that they are not always adhered to and may be insufficient for the purposes of encouraging researchers to report results in a way that makes them easily accessed and reused by others. One consequence of spending so many hours reviewing papers is that reviewers can become exhausted and may be prone to mistakes, or run out of time and motivation to complete the review [2]. In addition, with better reporting, much of this time and money could have been spent on arguably more important aspects of the research including analysis, write-up and publication of findings. Although ours is only a case study of one systematic review, we anticipate that poor reporting is leading to similar problems with other reviews, creating more avoidable research waste.

More disappointing and wasteful than reviewing papers not fitting inclusion criteria, was the number of studies that had used EQ-5D but could not be included in our meta-analysis. We found that only 60 from 130 papers reporting use of the EQ-5D could be used in the meta-analysis. More than half of the papers we found that had collected potentially useful data could not be included, which was over 130,000 participants. Adding to this, we may also have missed papers that did include useful data for our meta-analysis, by inadvertently excluding them at title and abstract screening, if it appeared that HRQOL had not been measured.

This second form of waste, due to inability to reuse results, is most problematic because as Chan et al. [21] point out, inability to access some research can lead to waste in the form of redundant studies about similar treatments, and harm through reliance on biased meta-analyses. A paper by Garcia-Alamino et al. [22] has shown that this problem is widespread and many meta-analyses are underpowered and subject to substantial uncertainty. It is difficult to quantify the effect of that waste, but the opportunity cost to potentially inform policy and clinical practice could be much more severe than a few days or weeks of wasted researcher time. For our meta-analysis, we know that we are missing about half of the data points that might otherwise have been included. This could have implications for our understanding of the evidence of effectiveness and cost-effectiveness of health services, and have serious consequences when such evidence is used to inform decisions about healthcare resource allocation. Health utility data are essential to understanding the value of different health services, but if estimates of health utility are biased, our estimates of value will also be flawed [21]. Whilst a limitation is that our findings apply specifically to coronary artery disease research, we expect research for other diseases to have similar levels of waste and therefore that these figures are generalisable. Given the time taken for only one disease, it would be impractical to undertake a review of this nature across all disease areas.

Similarly, a potential criticism is that we only searched for studies which had measured EQ-5D directly from participants, when it is possible to map EQ-5D outcomes from other instruments. Whilst we will have excluded studies which took that approach, there is debate about the reliability of mapping algorithms particularly for lower health utility values [23]. Therefore, for the purposes of the meta-analysis we are undertaking, we felt it was appropriate to exclude studies which mapped outcomes from other instruments to EQ-5D.

Despite substantial resources being invested to catalogue papers using Medical Subject Headings (MeSH) [24] terms and keywords, the only relevant MeSH term for this research is *Quality-of-life*, catalogued under both *Epidemiological measurements* and *Philosophy*. There is a MeSH term for *Patient-Reported Outcome Measures* (PROMs), but the only instrument listed is a sino-nasal outcome test for rhinosinusitis. A solution would be to include a more complete list of validated PROMs in the MeSH terms that would enable papers to be classified and more easily retrieved by researchers. The Australian Commission on Safety and Quality in Health Care has collated lists of all validated PROMs [25], which could be used as a starting point to update the MeSH terms.

In summary, the problem of poor reporting of HRQOL data can create research waste both from the perspective of researcher time spent retrieving and screening papers, and for the reliability meta-analysis results. The solutions we recommend are simple to implement and should improve both our ability to find previous research and our understanding of the value of treatments to patients through more reliable meta-analyses.

## Recommendations

- New MeSH terms for all validated PROMs to be proposed to the National Library of Medicine.
- Improve use of reporting guidance such as CONSORT-PRO and SPIRIT-PRO Extensions [10, 11] to ensure researchers include a list of PROMs collected in a study, in the abstract wherever possible. Author guidelines and administrative checks by journals may be potential mechanisms/forcing functions to ensure better reporting.
- Further extension of the guidelines to include improved reporting of EQ-5D data, to enable reuse: mean health utility weights, with standard deviation or standard error, number of people in the sample, and the tariff used (which country/region) to generate utility weights.
- Encourage researchers to use and report EQ-5D questionnaire results and avoid reporting only the Visual Analogue Scale (EQ VAS), which is not a preference-based measure.

## Data Availability

Data related to the systematic review and calculations of waste available on https://osf.io/uayh9/
